# Periprosthetic Joint Infection in Patients With Arthroplasty Undergoing Perioperative Colonoscopy

**DOI:** 10.1001/jamanetworkopen.2024.10123

**Published:** 2024-05-07

**Authors:** Ashley B. Anderson, Sean E. Slaven, Nora L. Watson, John P. Cody, Robert J. McGill, Benjamin K. Potter, Matthew D. Nealeigh

**Affiliations:** 1 Uniformed Services University–Walter Reed Department of Surgery, Walter Reed National Military Medical Center, Bethesda, Maryland; 2 Alexander T. Augusta Military Medical Center, Fort Belvoir, Virginia

## Abstract

**Importance:**

Periprosthetic joint infection (PJI) is a rare but devastating complication. Most patients undergoing total joint arthroplasty (TJA) also need routine screening colonoscopy, in which transient bacteremia may be a potential source for hematogenous PJI. Patients and surgeons must decide on an optimal time span or sequence for these 2 generally elective procedures, but no such guidelines currently exist.

**Objective:**

To evaluate associations of colonoscopy with the risk of post-TJA PJI for the development of clinical practice recommendations for colonoscopy screening in patients undergoing TJA.

**Design, Setting, and Participants:**

This retrospective cohort study of Military Health System (MHS) beneficiaries older than 45 years who underwent TJA from January 1, 2010, to December 31, 2016, used propensity score matching and logistic regression to evaluate associations of colonoscopy with PJI risk. Statistical analyses were conducted between January and October 2023.

**Exposure:**

Colonoscopy status was defined by *Current Procedural Terminology* code for diagnostic colonoscopy within 6 months before or 6 months after TJA.

**Main Outcomes and Measures:**

Periprosthetic joint infection status was defined by a PJI *International Classification of Diseases* code within 1 year after TJA and within 1 year from the post-TJA index colonoscopy date.

**Results:**

Analyses included 243 671 patients (mean [SD] age, 70.4 [10.0] years; 144 083 [59.1%] female) who underwent TJA in the MHS from 2010 to 2016. In the preoperative colonoscopy cohort, 325 patients (2.8%) had PJI within 1 year postoperatively. In the postoperative colonoscopy cohort, 138 patients (1.8%) had PJI within 1 year from the index colonoscopy date. In separate analyses of colonoscopy status within 6 months before and 6 months after TJA, younger age, male sex, and several chronic health conditions (diabetes, kidney disease, and pulmonary disease) were each associated with higher PJI risk. However, no association was found with PJI risk for perioperative colonoscopy preoperatively (adjusted odds ratio, 1.10; 95% CI, 0.98-1.23) or postoperatively (adjusted odds ratio, 0.90; 95% CI, 0.74-1.08).

**Conclusions and Relevance:**

In this large retrospective cohort of patients undergoing TJA, perioperative screening colonoscopy was not associated with PJI and should not be delayed for periprocedural risk. However, health conditions were independently associated with PJI and should be medically optimized.

## Key Points

**Question:**

Does preoperative or postoperative colonoscopy increase the risk of periprosthetic joint infection (PJI) in total joint arthroplasty?

**Findings:**

This cohort study of 243 671 Military Health System beneficiaries who underwent total joint arthroplasty in 2010 to 2016 found no association of preoperative or postoperative colonoscopy with PJI risk through 1-year follow-up. Several comorbid conditions were associated with PJI risk.

**Meaning:**

These findings suggest that colonoscopy within 6 months before or after total joint arthroplasty is not associated with increased postsurgery PJI risk.

## Teaser

This cohort study examines the incidence of periprosthetic joint infection in patients undergoing total hip arthroplasty and total knee arthroplasty and the associations of preoperative and postoperative colonoscopy with periprosthetic joint infection.

## Introduction

The number of patients undergoing total joint arthroplasty (TJA) of the hip and knee continues to increase as the US population ages.^1^ Published projections forecast an increase of 176% by 2040 and 659% by 2060 for total hip arthroplasty (THA) and 139% by 2040 and 469% by 2060 for total knee arthroplasty (TKA).^2^ With the increase in arthroplasty procedures, an increase in associated complications is likely.

Periprosthetic joint infection (PJI) after TJA procedures is a rare but devastating complication that is associated with increased morbidity and mortality. Despite research efforts, PJI risk is not improving over time, and projections and epidemiology of revision TJA demonstrate that the incidence of PJI is expected to increase proportionately with the demand for THA and TKA. This anticipated trend will severely impact morbidity and mortality because the 5-year overall survival proportions are 67% and 72% for PJI after THA and TKA, respectively, in the Medicare population.^3^

Likewise, with the aging US population, more patients will require routine screening procedures for general health maintenance. The US Multi-Society Task Force on Colorectal Cancer, which represents the American College of Gastroenterology, the American Gastroenterological Association, and the American Society for Gastrointestinal Endoscopy, recommends routine colonoscopy screening for colorectal cancer beginning at 45 years of age.^4^ Although the frequency of transient bacteremia in routine screening colonoscopy is low, transient bacteremia may be a potential source of hematogenous PJI in select patients. In a matched case-control study of patients undergoing TKA, the postoperative colonoscopy cohort had increased PJI risk at 9 months and 1 year after postoperative colonoscopy.^5^ Colonoscopy was associated with an increased PJI risk in TKA recipients, regardless of concomitant invasive colonoscopy procedures.^5^ In a subsequent study, antibiotic prophylaxis did not decrease 90-day through 1-year PJI risk in the setting of preexisting TKA. In adjusted analyses, colonoscopy cohorts were not associated with increased risk of PJI compared with TKA recipients who did not undergo subsequent colonoscopy.^6^ However, these publications were focused exclusively on TKA and postoperative colonoscopy. The American Academy of Orthopaedic Surgeons does not have a clear consensus statement for timing of colonoscopy because there is an unclear risk of PJI from transient bacteremia in accordance with the American Society for Gastrointestinal Endoscopy (ASGE) 2015 practice guidelines.^7^

When faced with scheduling colonoscopy for colorectal cancer screening with either an existing or upcoming hip or knee arthroplasty, patients and their orthopedic surgeons must decide whether there is an optimal period within which to schedule these 2 generally elective procedures and in which order. However, there are currently no perioperative guidelines to counsel patients on time from or time to routine colonoscopy in patients undergoing THA or TKA. The purpose of this retrospective cohort study was to characterize the PJI incidence in patients undergoing THA and TKA and the associations of colonoscopy before and after surgery with PJI risk during 1-year follow-up.

## Methods

### Guidelines and Data Source

This retrospective cohort study followed the Reporting of Studies Conducted Using Observational Routinely Collected Health Data (RECORD),^8^ an extension of the Strengthening the Reporting of Observational Studies in Epidemiology (STROBE) for reporting both an accurate and complete study (Figure).^9^ The Military Data Repository (MDR) contains comprehensive records of all health care encounters, including patient demographic characteristics and diagnosis and procedure codes, occurring within the Military Health System (MHS) at military treatment facilities or in the civilian sector and paid for by TRICARE. The MHS is considered a universal health care system for all service members, including active duty members, guards and reservists, retirees, and their family members. Its mission is to ensure the health and readiness of military service members by providing high-quality accessible care to its 9.5 million beneficiaries worldwide.

**Figure.**
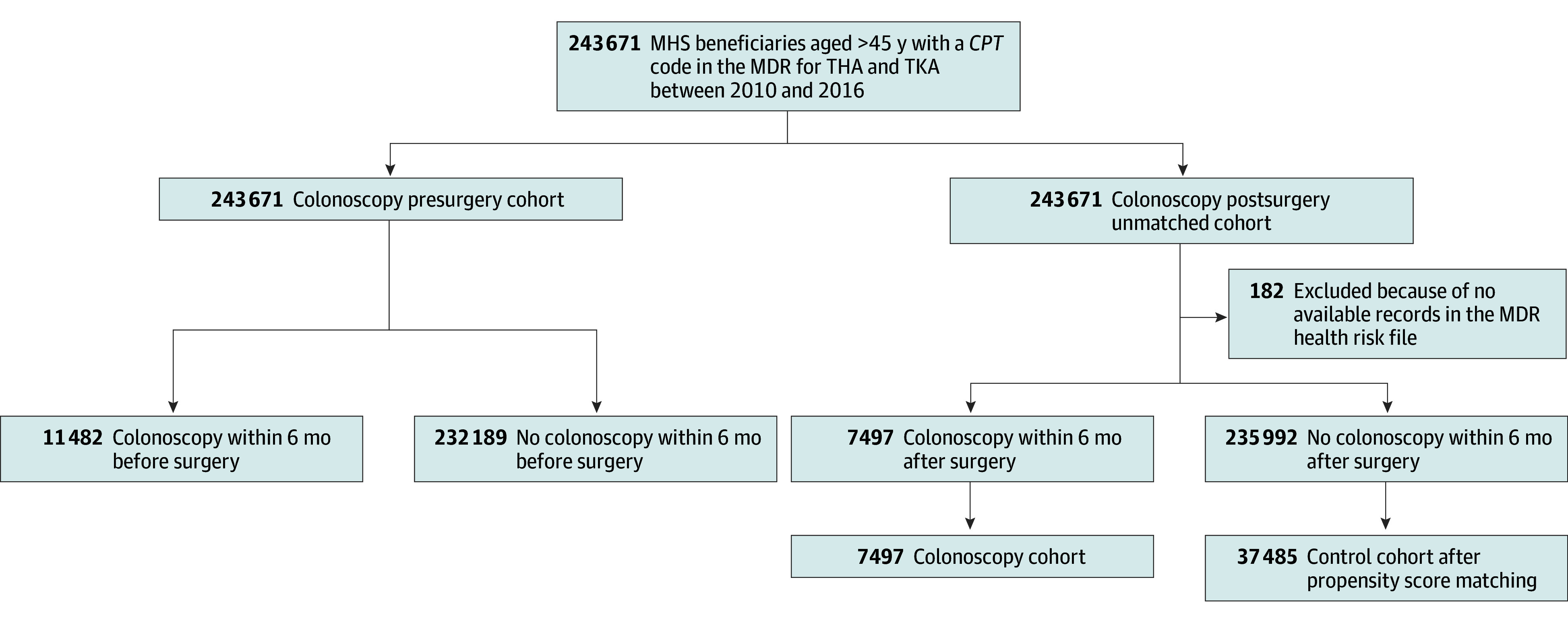
Study Flowchart *CPT* indicates *Current Procedural Terminology*; MDR, Military Data Repository; MHS, Military Health System; THA, total hip arthroplasty; and TKA, total knee arthroplasty.

### Patient Selection

After determination by the Walter Reed National Military Medical Center that the project was exempt from institutional review board approval and the need for informed consent in accordance with 32 *Code of Federal Regulations* §219.104(d), we queried the MDR for MHS beneficiaries older than 45 years who had *Current Procedural Terminology* (*CPT*) codes (27130, 27132, 27125, 27236, 27134, 27137, 27138, 27091, 27090, 27440, 27441, 27442, 27443, 27445, 27446, 27447, 27486, and 27487) for hip or knee joint arthroplasty from January 1, 2010, to December 31, 2016, in either a military treatment facility or the civilian health care system. Records in the query were linked for analysis by a nonidentifying pseudo–identification number and included no personally identifiable information. Follow-up data were limited to through 2018 because substantial changes in MDR data fields and their pending completion, present at the time of analysis in 2023, resulted from the MHS’s transition to a new electronic health record system largely beginning in 2019.

### Study Design and Variables

Descriptive statistics were reported for demographic characteristics, including age, sex, and beneficiary status (active duty, retirees, and dependents of active duty or all others [primarily dependents of retirees]) and relevant comorbidities, stratified by colonoscopy status. Because patient race and ethnicity were frequently not reported in the MDR, these data were not included in analyses. Comorbidities were defined by record of the health condition in the MDR health risk file, representing presence of the health comorbidities stratified by physiologic system, in the year of TJA. Characteristics of the study cohort are given in Table 1. Colonoscopy status preoperatively was identified by the presence of a colonoscopy *CPT* code within 6 months before TJA. In separate analyses, we identified colonoscopy postoperatively by the presence of a colonoscopy *CPT* code within 6 months after TJA. Patients were excluded from adjusted analyses if they did not have an available record in the health risk file in the year of surgery (n = 182). All patients had available demographic characteristics (age, sex, and beneficiary status) at the surgery encounter. To address the potential for misclassification of colonoscopy status in Medicare-eligible patients, for whom TRICARE is generally a second payer, analyses of both the preoperative and postoperative cohorts were repeated among the subgroups of patients younger than 65 years to evaluate for changes in colonoscopy and event frequencies and model estimates that may suggest bias due to censoring of diagnostic colonoscopies that were paid fully by Medicare.

**Table 1.** Demographic and Clinical Characteristics of the Presurgery Cohort^a^

Characteristic	Colonoscopy (n = 11 482)^b^	No colonoscopy (n = 232 189)	All (N = 243 671)
Age, mean (SD), y	69.0 (9.0)	70.5 (10.1)	70.4 (10.0)
Sex			
Female	6538 (56.9)	137 545 (59.2)	144 083 (59.1)
Male	4944 (43.1)	94 644 (40.8)	99 588 (40.9)
Beneficiary status			
Active duty	138 (1.2)	2130 (0.9)	2268 (0.9)
Dependents or others	6410 (55.9)	135 860 (58.5)	142 270 (58.4)
Retired	4934 (43.0)	94 199 (40.6)	99 133 (40.7)
No. of chronic conditions, mean (SD)	4.00 (1.46)	3.37 (1.49)	3.40 (1.50)
Cerebrovascular disease	1038 (9.0)	19 988 (8.6)	21 026 (8.6)
Cardiovascular disease	10 042 (87.5)	189 025 (81.5)	199 067 (81.8)
Diabetes	3216 (28.0)	58 306 (25.1)	61 522 (25.3)
Kidney disease	4236 (36.9)	78 720 (33.9)	82 956 (34.1)
Cancer	7142 (62.2)	55 194 (23.8)	62 336 (25.6)
Pulmonary disease	5701 (49.7)	102 296 (44.1)	107 997 (44.4)

^a^ Data are presented as number (percentage) of participants unless otherwise indicated.

^b^ Colonoscopy status was defined by *International Classification of Diseases, Ninth Revision *or *International Statistical Classification of Diseases and Related Health Problems, Tenth Revision *code for diagnostic colonoscopy present within 6 months before surgery.

### Outcomes of Interest

The primary outcomes were incidences of PJI within 1 year after TJA (preoperative colonoscopy cohort) and 1 year from post-TJA index colonoscopy date (postoperative colonoscopy cohort). The PJI status was defined by presence of relevant PJI *International Classification of Diseases, Ninth Revision* (*ICD-9*) and *International Statistical Classification of Diseases and Related Health Problems, Tenth Revision* (*ICD-10*) codes (996.69, 996.60, 996.66, 996.67, T84.50XA, T84.50XD, T84.50XS, T84.59XA, T84.59XD, T84.59XS, T84.53XA, T84.53XD, T84.53XS, T84.54XA, T84.54XD, and T84.54XS). Additionally, risk factors for PJI were investigated in both the preoperative and postoperative colonoscopy cohorts.

### Statistical Analysis

#### Preoperative Colonoscopy

Multivariable logistic regression was used to estimate adjusted odds ratios (AORs) associated with preoperative colonoscopy status as the independent variable, with demographic and chronic conditions as confounding risk factors, and PJI within 1 year after TJA as the dependent variable. Additional analyses defined colonoscopy presence within 3 and 12 months before TJA and stratified primary analyses by TJA type (hip vs knee) to evaluate for sensitivity of estimates to the time between procedures or surgery type.

#### Postoperative Colonoscopy

Propensity score matching was used to minimize risks of both confounding and ascertainment bias particular to the postoperative setting, in which patients who did vs did not have a colonoscopy ascertained after surgery may be expected to have longer available follow-up for postoperative PJI.^10^ Propensity score–matched colonoscopy (case) and noncolonoscopy (control) cohorts, balanced by baseline demographic and clinical characteristics considered as confounders, were defined using nearest neighbor matching with a 1:5 case-control ratio. Propensity scores for probability of colonoscopy after TJA as the dependent variable were estimated with a logistic regression model of age, sex, beneficiary status, TJA surgery year, and presence of any health risk condition as independent variables. Balance in characteristics among matched cohorts was evaluated with standardized mean differences. A matched colonoscopy index date was assigned from each case to that case’s matched controls. The PJI status was then identified by PJI *ICD-9* or *ICD-10* code presence within 1 year after the colonoscopy index date. Multivariable logistic regression was used to evaluate AORs associated with colonoscopy status and demographic and health risk characteristics as independent variables and PJI within 1 year of the post-TJA index colonoscopy date as the dependent variable. Sensitivity analyses repeated matched cohorts and OR estimates when defining colonoscopy postoperatively within 12 months after TJA. Propensity score matching was performed using the matchit package in R, version 4.0.5 (R Foundation for Statistical Computing).^11^ Statistical significance was defined for all analyses as a 2-sided *P* < .05 with additional sensitivity analyses interpreted as exploratory. Statistical analyses were conducted between January and October 2023.

## Results

### Preoperative Colonoscopy

Analyses of PJI risk associated with preoperative colonoscopy included 243 671 patients older than 45 years who had TJA in the MHS from 2010 to 2016 (Table 1). Mean (SD) age of the cohort was 70.4 (10.0) years; 144 083 patients (59.1%) were women and 99 588 (40.9%) were men. Of these patients, 11 482 (4.7%) received a colonoscopy within 6 months before surgery. The risk of PJI within 1 year postoperatively was 2.8% (n = 325) in patients who did vs 2.4% (n = 5504) in patients who did not have a colonoscopy within 6 months before surgery (Table 2) (unadjusted OR, 1.20; 95% CI, 1.07-1.34).

**Table 2.** Characteristics of the Presurgery Cohort by Joint Infection Presence Within 1 Year After Surgery^a^

Characteristic	PJI (n = 5829)	No PJI (n = 237 842)	AOR (95% CI)^b^
Age, mean (SD), y	70.0 (10.2)	70.4 (10.0)	0.85 (0.83-0.87)
Sex			
Female	3120 (2.2)	140 963 (97.8)	1.30 (1.23-1.37)
Male	2709 (2.7)	96 879 (97.3)	
Beneficiary status			
Active duty	42 (1.8)	2226 (98.1)	0.69 (0.50-0.93)
Dependents or others	3125 (2.2)	139 145 (58.5)	
Retired	2662 (2.7)	96 471 (97.3)	
No. of chronic conditions, mean (SD)	3.96 (1.56)	3.39 (1.49)	NA
CBVD	641 (3.0)	20 385 (97.0)	1.12 (1.03-1.22)
CVD	4994 (2.5)	194 073 (97.5)	1.09 (1.01-1.18)
Diabetes	1905 (3.1)	59 617 (96.9)	1.26 (1.19-1.33)
Kidney disease	2802 (3.4)	80 154 (96.6)	1.73 (1.64-1.83)
Cancer	1595 (2.6)	60 741 (97.4)	1.00 (0.94-1.07)
Pulmonary disease	3361 (3.1)	104 636 (96.9)	1.59 (1.51-1.68)
Colonoscopy before surgery			
<3 mo	157 (2.7)	5578 (97.3)	1.06 (0.90-1.24)
<6 mo	325 (2.8)	11 157 (97.2)	1.10 (0.98-1.23)
<12 mo	583 (2.6)	21 603 (97.4)	1.02 (0.93-1.11)

Abbreviations: AOR, adjusted odds ratio; CBVD, cerebrovascular disease; CVD, cardiovascular disease; NA, not applicable; PJI, periprosthetic joint infection.

^a^ Data are presented as number (row percentage) of participants unless otherwise indicated.

^b^ The AORs for age were estimated per 10 years older age. The AORs were estimated by a multivariable logistic regression model of characteristics and colonoscopy less than 6 months before surgery as independent variables and joint infection within 1 year after surgery as the dependent variable. The AORs for colonoscopy at less than 3 months and less than 12 months are estimated by the model repeated to define colonoscopy presence as within 3 months or 12 months before surgery. The AOR for sex is male vs female, and the AOR for beneficiary status is active duty vs dependents and others or retired as reference.

In a multivariable regression model of demographic and health risk characteristics and colonoscopy status as factors associated with PJI within 1 year of TJA, active duty status (AOR, 0.69; 95% CI, 0.50-0.93) and older age (AOR, 0.85; 95% CI, 0.83-0.87 per 10-year increase) were each associated with lower PJI risk. Male sex (AOR, 1.30; 95% CI, 1.23-1.37) and several chronic health conditions (cerebrovascular disease, cardiovascular disease, diabetes, kidney disease, and pulmonary disease) were associated with higher PJI risk (Table 2).

Colonoscopy within 6 months before surgery was not independently associated with PJI risk (AOR, 1.10; 95% CI, 0.98-1.23). The AOR for colonoscopy was also not statistically significant when colonoscopy presence was defined in separate analyses within up to 1 year before (AOR, 1.02; 95% CI, 0.93-1.11) and within 3 months before surgery (AOR, 1.06; 95% CI, 0.90-1.24). When analyses were repeated among subgroups defined by TJA type (hip vs knee), the risk of PJI in patients by colonoscopy status and AORs was similar to results in the full cohort.

In sensitivity analyses restricted to patients younger than 65 years, 3974 patients (5.2%) had a diagnostic colonoscopy within 6 months before surgery. Model estimates for this exposure did not substantially change, with the exception of increased risk of PJI associated with prevalent cancer (AOR, 1.13; 95% CI, 1.00-1.26).

### Postoperative Colonoscopy

Postoperative analyses included 7497 patients who had a colonoscopy within 6 months after TJA and 37 485 matched controls with TJA who did not have a colonoscopy (Table 3). The risk of PJI within 1 year of the post-TJA index colonoscopy date was 1.8% (n = 138) in the colonoscopy vs 2.1% (n = 788) in the control cohort (Table 4).

**Table 3.** Demographic and Clinical Characteristics of Postsurgery Cohorts Before and After Propensity Score Matching^a^

Characteristic	Unmatched cohort		SMD	Matched cohort		SMD
	Colonoscopy cohort (n = 7497)	Noncolonoscopy cohort (n = 235 992)		Colonoscopy cohort (n = 7497)	Noncolonoscopy cohort (n = 37 485)	
Age, mean (SD), y	69.7 (9.1)	70.5 (10.1)	0.09	69.7 (9.1)	69.8 (9.0)	0.02
Sex						
Female	4118 (54.9)	139917 (59.3)	0.09	4118 (54.9)	20418 (54.5)	0.01
Male	3379 (45.1)	96075 (40.7)		3379 (45.1)	17067 ( 45.5)	
Beneficiary status						
Active duty	107 (1.4)	2160 (0.9)	0.04	107 (1.4)	393 (1.0)	0.03
Dependents or others	4036 (53.9)	138067 (58.5)	0.09	4036 (53.9)	20148 (53.7)	0.00
Retired	3354 (44.7)	95765 (40.6)	0.08	3354 (44.7)	16944 (45.2)	0.01
No. of chronic conditions, mean (SD)	3.9 (1.5)	3.4 (1.5)	0.33	3.9 (1.5)	3.9 (1.5)	0.01
Year of surgery						
2010	1277 (17.0)	35992 (15.3)	0.05	1277 (17.0)	6396 (17.1)	0.00
2011	1155 (15.4)	33380 (14.1)	0.03	1155 (15.4)	5798 (15.5)	0.00
2012	1051 (14.1)	33518 (14.2)	0.01	1051 (14.1)	5268 (14.1)	0.00
2013	1082 (14.4)	33176 (14.1)	0.01	1082 (14.4)	5355 (14.3)	0.00
2014	1041 (13.9)	32762 (13.9)	0.00	1041 (13.9)	5190 (13.8)	0.00
2015	972 (13.0)	33059 (14.0)	0.03	972 (13.0)	4894 (13.1)	0.00
2016	919 (12.3)	34105 (14.5)	0.07	919 (12.3)	4584 (12.2)	0.00

Abbreviation: SMD, standardized mean difference.

^a^ Data are presented number (percentage) of participants unless otherwise indicated.

**Table 4.** AORs for Joint Infection Within 1 Year After Surgery in Postsurgery Cohorts^a^

Characteristic	Joint infection (n = 926)	No joint infection (n = 44 056)	AOR (95% CI)^b^
Age (AOR per 10 y), mean (SD), y	69.8 (8.7)	69.8 (9.0)	0.89 (0.82-0.96)
Sex			
Female	465 (1.9)	24 071 (98.1)	1.23 (1.08-1.40)
Male	461 (2.2)	19 985 (97.7)	
Active duty	6 (1.2)	494 (98.8)	0.61 (0.24-1.26)
No. of chronic conditions, mean (SD)	4.52 (1.49)	3.89 (1.53)	NA
CBVD	138 (2.8)	4850 (97.2)	1.20 (0.99-1.44)
CVD	829 (2.1)	38 201 (97.9)	0.98 (0.79-1.23)
Diabetes	398 (2.8)	14 031 (97.2)	1.41 (1.23-1.62)
Kidney disease	507 (2.7)	17 995 (97.3)	1.57 (1.36-1.80)
Cancer	344 (2.2)	15 345 (97.8)	1.05 (0.92-1.21)
Pulmonary disease	610 (2.6)	22 768 (97.4)	1.63 (1.41-1.88)
Index colonoscopy after surgery			
<6 mo	138 (1.8)	7359 (98.2)	0.90 (0.74-1.08)
<12 mo	283 (1.5)	18 800 (98.5)	0.83 (0.65-1.04)

Abbreviations: AOR, adjusted odds ratio; CBVD, cerebrovascular disease; CVD, cardiovascular disease; NA, not applicable.

^a^ Data are presented as number (row percentage) of participants unless otherwise indicated.

^b^ The AORs were estimated from a multivariable logistic regression model of characteristics and index colonoscopy less than 6 months after surgery as independent variables and joint infection within 1 year after the index colonoscopy date as the dependent variable in propensity score–matched cohorts. Matched cohorts were defined by colonoscopy status within 6 months after surgery. The AOR for sex is male vs female, and the AOR for beneficiary status is active duty vs dependents or others or retired as reference.

Before propensity score matching, patients undergoing TJA who had colonoscopy within 6 months postoperatively were more likely to be male and retirees and had a higher number of comorbidities and an earlier year of surgery (Table 3). After propensity score matching, frequencies of categorical covariates were similar between cohorts, and standardized mean differences were near 0, indicating balance in the distributions of these confounders.

In a multivariable logistic regression model of demographic and health risk characteristics and colonoscopy status as factors associated with postoperative PJI, older age (AOR, 0.89; 95% CI, 0.82-0.96 per 10-year increase) was associated with lower PJI risk (Table 4). Male sex (AOR, 1.23; 95% CI, 1.08-1.40) and several comorbidities (eg, diabetes, kidney disease, and pulmonary disease) were associated with higher PJI risk (Table 4). Colonoscopy within 6 months postoperatively was not associated with PJI risk (AOR, 0.90; 95% CI, 0.74-1.08). This AOR was similar when defining colonoscopy within 12 months postoperatively. In sensitivity analyses of colonoscopy 6 months postoperatively as the exposure in the subgroup of patients younger than 65 years, AORs for age, male sex, and diabetes were no longer statistically significant and did not substantially change for other patient characteristics. Frequencies of PJIs displayed by months from colonoscopy to surgery and months from index colonoscopy date to joint infection did not qualitatively suggest dependence of PJI risk on time between procedures.

## Discussion

In this large military beneficiary cohort with universal health care access, no independent association was found between colonoscopy and PJI risk through 1-year follow-up in patients who underwent preoperative or postoperative colonoscopy. Comorbid conditions, including kidney and pulmonary disease, diabetes, and inflammatory conditions, were associated with significant PJI risk. This is the first study, to our knowledge, to report these findings for both TKA and THA patient populations preoperatively and postoperatively.

The overall risks of PJI in the preoperative and postoperative analysis cohorts were 2.4% and 2.1%, respectively. Analyses of both cohorts suggest that transient bacteremia from preoperative or postoperative colonoscopy does not increase the risk of PJI for TJA. Although colonoscopy within 6 months before surgery was statistically associated with PJI risk in unadjusted analyses, the modest OR was attenuated and no longer significant when adjusted for confounding by the presence of chronic health conditions at the time of surgery and remained nonsignificant in sensitivity analyses for colonoscopy defined within 3 and 12 months before TJA. These sensitivity analyses were planned a priori because underlying confounding by unmeasured or misclassified characteristics are common limitations of retrospective analyses of health care databases, warranting caution when interpreting subtle effect sizes.

On the basis of these results, we do not recommend delaying TJA after colonoscopy for patients with joint pain that is limiting function and quality of life. Similarly, we do not recommend delaying routine health screening procedures, such as colonoscopy after TJA, because postoperative timing does not appear to increase the risk of PJI. However, these recommendations should be taken with caution when treating patients with select preoperative (eg, cerebrovascular disease, cardiovascular disease, diabetes, kidney disease, and pulmonary disease) and postoperative (eg, diabetes, kidney disease, and pulmonary disease) comorbidities that are independent risk factors for PJI in our study cohort. Our findings overlapped with previously published results^12,13^ on patient-related risk factors for PJI in THA and TKA cohorts. Bozic et al^12,13^reported rheumatologic disease, obesity, and preoperative anemia to be risk factors for PJI after THA^12^ as well as congestive heart failure, chronic pulmonary disease, preoperative anemia, diabetes, depression, kidney disease, pulmonary circulation disorders, obesity, rheumatologic disease, psychoses, metastatic tumor, peripheral vascular disease, and valvular disease to be risk factors for PJI after TKA.^13^ In these higher-risk patient populations, it is important to weigh the risk and benefits of timing between elective arthroplasty and completion of routine health screening procedures, specifically preoperatively. Additionally, in select high-risk patients undergoing colonoscopy after TJA, it may be important to consider periprocedural antibiotic prophylaxis for infection because of the risk, although low, of transient bacteremia from mechanical manipulation of the colon, which has a large bacterial colony burden.^14^ However, this decision-making process should be shared among the patient, gastroenterologist, and orthopedic surgeon, as the use of antibiotic prophylaxis does not decrease PJI risk after colonoscopy in TKA^6^ and the ASGE Standards of Practice Committee recommends against routine antibiotics.^7^

Interestingly, our incidences of PJI in the control group were higher than those previously reported in the literature.^2,3,15,16,17^ The mean age of our cohort was 70.4 years, with preoperative and postoperative rates of 2.4% and 2.1%, respectively. Using Medicare data from 2005 to 2015, Kurtz et al^3^ showed that the 1-year and 5-year risks of PJI are not decreasing despite a focus on infection prevention. The rate of PJI ranges from 0.5% to 2.0% for TKA and 0.2% to 1.0% for THA.^15,16,17^ Our higher incidences from 2010 to 2016 over similar follow-up may be a reflection of stable PJI rates but an increasing number of procedures performed, just as we would expect the incidence of PJI to increase proportionately with the demand for TJA.^2^

### Limitations

This study has several limitations. This study was retrospective in design, and the potential for diagnosis and procedure coding errors and unmeasured confounding are limitations that can occur with retrospective analyses of large administrative databases. However, we followed the STROBE reporting guidelines, and the risk of confounding by indication was mitigated by our propensity score–matched approach to achieve balance in known confounders, including the timing of the index colonoscopy. Importantly, MDR analyses that include patients 65 years or older must consider risk of bias due to censoring of procedures that were fully paid for by Medicare. Our sensitivity analyses restricted to patients younger than 65 years were generally consistent with findings in the full cohort, suggesting that risk of informative censoring may be mitigated by our inclusion of patients who had TJA either partially or fully paid for by TRICARE. Additionally, the (expected) low incidence of PJI in the patient population, despite our large cohort, prevented our ability to evaluate for potential variation in risk in patients who had biopsy or other endoscopy-related invasive procedures or to formally characterize potential differences in risk with colonoscopy exposures less than 6 months preoperatively and postoperatively. This limitation may obscure a potential elevated PJI risk associated with colonoscopy exposure defined as less than 1 to 2 months after TJA^18^; this association, however, was not adjusted for individual comorbidities that were associated with both colonoscopy and PJI in the current and previous cohorts. We also limited our follow-up period for PJI to 1 year after surgery or postsurgery colonoscopy, which is generally consistent with the defined period of surgical site infections with underlying implants but would exclude the rare cases of late PJI.^19^

## Conclusions

Timing of colonoscopy before or after TJA was not associated with increased PJI risk 1 year after surgery in this MHS population. However, several comorbidities were independently associated with PJI, and they should be medically optimized before and after TJA. These data build on evidence from large retrospective cohorts that may be used to guide the development of clinical practice recommendations to better counsel patients undergoing TJA and coordinate scheduling and prioritization between these 2 generally elective but important procedures.

## Notes

Supplement.
Data Sharing Statement
