# Supplementary material for: Periprosthetic Joint Infection in Patients With Arthroplasty Undergoing Perioperative Colonoscopy
Source: JAMA Netw Open. 2024 May 7;7(5):e2410123. doi: 10.1001/jamanetworkopen.2024.10123 (PMC11077397; doi:10.1001/jamanetworkopen.2024.10123)
Supplement: Supplement. — Data Sharing Statement [file jamanetwopen-e2410123-s001.pdf]

Data Sharing Statement

Anderson. Periprosthetic Joint Infection in Patients With Arthroplasty Undergoing Perioperative Colonoscopy. *JAMA Netw Open*. Published May 07, 2024.
doi:10.1001/jamanetworkopen.2024.10123

Data
Data available: No

Additional Information
Explanation for why data not available: The data is military data that can be accessible with data sharing agreements.
